# Farnesyl Phosphatase, a *Corpora allata* Enzyme Involved in Juvenile Hormone Biosynthesis in *Aedes aegypti*

**DOI:** 10.1371/journal.pone.0071967

**Published:** 2013-08-05

**Authors:** Pratik Nyati, Marcela Nouzova, Crisalejandra Rivera-Perez, Mark E. Clifton, Jaime G. Mayoral, Fernando G. Noriega

**Affiliations:** Department of Biological Sciences, Florida International University, Miami, Florida, United States of America; Centro de Pesquisas René Rachou, Brazil

## Abstract

**Background:**

The juvenile hormones (JHs) are sesquiterpenoid compounds that play a central role in insect reproduction, development and behavior. The late steps of JH III biosynthesis in the mosquito *Aedes aegypti* involve the hydrolysis of farnesyl pyrophosphate (FPP) to farnesol (FOL), which is then successively oxidized to farnesal and farnesoic acid, methylated to form methyl farnesoate and finally transformed to JH III by a P450 epoxidase. The only recognized FPP phosphatase (FPPase) expressed in the *corpora allata* (CA) of an insect was recently described in *Drosophila melanogaster* (*Dm*FPPase). In the present study we sought to molecularly and biochemically characterize the FPP phosphatase responsible for the transformation of FPP into FOL in the CA of *A. aegypti*.

**Methods:**

A search for orthologs of the *Dm*FPPase in *Aedes aegypti* led to the identification of 3 putative FPPase paralogs expressed in the CA of the mosquito (*Aa*FPPases-1, -2, and -3). The activities of recombinant *Aa*FPPases were tested against general phosphatase substrates and isoprenoid pyrophosphates. Using a newly developed assay utilizing fluorescent tags, we analyzed *Aa*FPPase activities in CA of sugar and blood-fed females. Double-stranded RNA (dsRNA) was used to evaluate the effect of reduction of *Aa*FPPase mRNAs on JH biosynthesis.

**Conclusions:**

*Aa*FPPase-1 and *Aa*FPPase-2 are members of the NagD family of the Class IIA C2 cap-containing haloalkanoic acid dehalogenase (HAD) super family and efficiently hydrolyzed FPP into FOL. *Aa*FPPase activities were different in CA of sugar and blood-fed females. Injection of dsRNAs resulted in a significant reduction of *Aa*FPPase-1 and *Aa*FPPase-2 mRNAs, but only reduction of *Aa*FPPase-1 caused a significant decrease of JH biosynthesis. These results suggest that *Aa*FPPase-1 is predominantly involved in the catalysis of FPP into FOL in the CA of *A. aegypti*.

## Introduction

Juvenile hormone (JH) plays a central role in insect development and reproduction [1]. JH is synthesized by the *corpora allata* (CA), a pair of endocrine glands connected to the brain [2]. The late steps of JH III biosynthesis in the mosquito *Aedes aegypti* involve the hydrolysis of farnesyl pyrophosphate (FPP) to farnesol (FOL), which is then successively oxidized to farnesal and farnesoic acid (FA) by an alcohol dehydrogenase [3] and an aldehyde dehydrogenase [4]. Farnesoic acid is methylated by a juvenile hormone acid methyl transferase [5] to form methyl farnesoate. In the last step, methyl farnesoate is transformed to JH III by a P450 epoxidase [6]. Characterization of CA enzymes has been hindered by the minute size of the endocrine gland; recently, the first description of an FPP phosphatase (FPPase) expressed in the CA of an insect was described in *Drosophila melanogaster* [7]. It is a member of the haloalkanoic acid dehalogenase (HAD) super family that catalyzes phosphoryl transfer reactions on a remarkably diverse set of substrates and includes enzymes such as: phosphoesterases, ATPases, phosphonatases, dehalogenases and sugar phosphomutases [8], [9]. HAD phosphatases employ an aspartate residue as a nucleophile in a magnesium-dependent phosphoaspartyl transferase reaction. The HAD superfamily is represented in the proteomes of organisms from all three super-kingdoms. The highly conserved structural core of the HAD enzymes consists of a α/β domain that adopts the topology typical of the Rossmann α/β folds housing the catalytic site and can be distinguished from all other Rossmanoid folds by two unique structural motifs: 1) an almost complete α-helical turn, named the ‘squiggle’, and 2) a β-hairpin turn, termed the ‘flap’ [10], [11]. The catalytic site is thus a composite of the four loops of the core domain and loop 5 of the cap domain. Whereas the core domain orchestrates the core chemistry, the cap domain functions in adapting that chemistry to a specific substrate (11).

The HAD superfamily can be divided into three generic classes based on the existence and location of a cap domain involved in substrate recognition. Class I possesses a small α-helical bundle cap between motifs I and II; Class II displays a cap between the second and third motifs; and Class III members present no cap domain [11]. Members of the HAD phosphatase superfamily have four conserved amino acid signature motifs [12], [9], [13]. These 4 signature motifs are also well conserved in the FPPase described in *Drosophila* (*Dm*FPPase) [7]. Bioinformatics searches for orthologs of the *Dm*FPPase in *A. aegypti* led to the identification of 3 putative FPPase paralogs expressed in the CA of the mosquito (*Aa*FPPase-1, -2, and -3). Recombinant *Aa*FPPase-1 and *Aa*FPPase-2 were found to efficiently hydrolyze FPP into FOL. Different FPPase activities were detected in CA extracts from adult female mosquitoes having diverse JH biosynthetic rates. Injection of dsRNAs resulted in a significant reduction of *Aa*FPPase-1 and *Aa*FPPase-2 mRNAs, but only reduction of *Aa*FPPase-1 caused a significant decrease on JH biosynthesis. These results suggest that *Aa*FPPase-1 is predominantly involved in the catalysis of FPP into FOL in the CA of *A. aegypti*.

## Materials and Methods

### 2.1. Chemicals

FPP, geranyl diphosphate (GPP) and isopentenyl diphosphate (IPP) were purchased from Echelon Biosciences (Salt Lake City, UT). p-nitrophenyl phosphate (*p-*NPP) was purchased from MP Biomedicals (Santa Ana, CA). N-acetyl-S-geranylgeranyl-L-cysteine (AGGC) and N-acetyl-S-farnesyl-L-cysteine (AFC) were purchased from Cayman chemicals (Ann Arbor, MI). Taurolithocholic acid 3-sulfate was purchased from Sigma-Aldrich (St. Louis, MO).

### 2.2. Insects

*A. aegypti* of the Rockefeller strain were reared at 28°C and 80% relative humidity under a photoperiod of 16 h light: 8 h dark. A cotton pad soaked in 3% sucrose solution was provided to adults. Four-day-old female mosquitoes were membrane-fed porcine blood equilibrated to 37°C, and ATP was added to the blood meal to a final concentration of 1 mM immediately before use.

### 2.3. Expression of recombinant AaFPPases

*Aa*FPPase cDNAs were expressed in *E. coli* cells as described by Mayoral *et al.* [5]. Recombinant His-tagged proteins were purified using HiTrap chelating columns and PD-10 desalting columns (Amersham Pharmacia, Piscataway, NJ). Glycerol was added to the enzyme solution (final concentration 50%), and samples were stored at −20°C until used. Protein concentrations were determined using the bicinchoninic acid protein assay reagent (BCA) (Pierce, Rockford, IL). Bovine serum albumin was used as a standard.

### 2.4. Enzyme assays

#### 2.4.1 Phosphatase assay

The catalytic activity of recombinant *Aa*FPPases towards *p*-NPP was measured in 96 well plates as described by Cao *et al.* [7]. Phosphatase activities towards different isoprenoid pyrophosphate substrates were determined using the Malachite Green Phosphate Assay Kit (Bioassay Systems, Hayward, CA); enzymatic activities were assayed using 40 µL reaction mixtures containing 100 mM MES, pH 6.0, 2 mM MgCl_2_, substrate (150 µM) and 75 ng of enzyme. After 20 min of incubation at 37°C, the reaction was terminated by the addition of the malachite green reagent (4∶1 v/v), and 30 min later the production of Pi was measured at 630 nm using a BioTek plate reader (BioTek, Winooski, VT). Kinetic parameters were determined by non-linear curve fitting using the GraphPad Prism software (San Diego, CA).

#### 2.4.2 RP-HPLC analysis of FPPase catalytic products

Production of FOL from FPP hydrolysis was analyzed by reverse-phase HPLC. FPP (250 µM) was incubated with recombinant *Aa*FPPase for 60 min in buffer (100 mM MES, pH 6.0, 2 mM MgCl_2_). Reactions were terminated by adding 500 µl of acetonitrile. Samples were centrifuged at 14,000 rpm for 5 min and the organic phase was recovered, filtered and analyzed by reverse-phase HPLC on a Dionex Summit System (Dionex, Sunnyvale, CA) equipped with a UVD 170U detector, 680 HPLC pump, TCC 100 column oven and Chromeleon software. HPLC was performed on an analytical column Acclaim 120 C18 (250×2.1 mm ID, particle size 5 µm) (Dionex), using isocratic elution from 0 to 20 min (acetonitrile/water, 1∶1 v/v), followed by a linear gradient from 20 to 50 min (acetonitrile-water (50 to 95%, v/v) and another isocratic elution from 50 min (acetonitrile, 95%). Flow rate was 0.2 ml/min and column temperature was 25°C. The eluate was monitored with UV (214 nm). Water or/and glycerol were used in place of recombinant enzymes in negative controls.

#### 2.4.3 Effect of inhibitors on AaFPPase activity

Recombinant *Aa*FPPases were pre-incubated with different concentrations (0 to 40 µM) of putative inhibitors for 10 min and their activities were measured using the *p*-NPP assay. The following compounds were tested: N-acetyl-S-geranylgeranyl-L-cysteine (AGGC), N-acetyl-S-farnesyl-L-cysteine (AFC) and taurolithocholic acid 3-sulfate.

### 2.5. Quantitative real-time PCR (qPCR)

RNA isolation and qPCR were performed as described by Nouzova et al. [6]. The primers and probes for the house keeping gene 60S ribosomal protein rpL32 and *Aa*FPPase transcripts are included in Table S1.

### 2.6. RNAi experiments

Synthesis and microinjections of double-stranded RNA (dsRNA) were performed as described by Perez-Hedo et al. [14]. *Aa*FPPases and YFP (yellow fluorescent protein) target sequences for dsRNA synthesis were amplified by PCR using the *Aa*FPPase-i and YFP-i primers (Table S1). The resulting amplicons were diluted 50-fold, and 1 µl was used as template in PCR reactions with primers containing T7 promoter sequences (Table S1). The products from these PCR reactions were purified using a QIAquick PCR purification kit (QIAquick sciences, Germantown, MD), and 1–2 µg of the purified DNA templates were used to synthesize dsRNAs with a Megascript RNAi kit (Ambion, Austin, TX). dsRNAs were precipitated using ammonium acetate/ethanol, and resuspended in ddH_2_O to a final concentration of 3–4 µg/µl. In each knockdown experiment, newly emerged female mosquitoes were cold anesthetized and injected intrathoracically with 1.6 µg of dsRNA using a Drummond Nanoject II microinjector and a micromanipulator. The effect of dsRNA was evaluated 4 days after injection, a time selected based on the analysis of dsRNA depletion experiments.

### 2.7. FPPase activity in CA extracts

FPPase activities in mosquito CA-CC (*corpora allata-corpora cardiaca* complex) were measured by HPLC coupled to a fluorescent detector (HPLC-FD) monitoring the production of farnesol. Glands were dissected in buffer solution (100 mM MES pH 6.0, 2 mM MgCl_2_). CA-CC were homogenized for 1 min, sonicated 3 min and centrifuged at 10,000 g for 10 min at 4°C. Supernatants were recovered and used as crude extract for activity assays as previously described [4]. The reaction products were labeled with DBD-COC1 for further quantification on HPLC-FD [15]. Controls such as boiled crude extract and reactions without enzyme were included. A standard curve was constructed for the quantification of tagged farnesol.

### 2.8. JH biosynthesis assay

The amount of JH synthesized by CA-CC complexes *in vitro* was quantified by high performance liquid chromatography coupled to a fluorescent detector (HPLC-FD) [15]. The assay is based on the derivatization of JH III with a fluorescent tag with subsequent analysis by reverse phase HPLC-FD.

### 2.9. Secondary structure and phylogenetic analysis

The secondary structure for *Aa*FPPase-1 was predicted using the protein structure homology-modeling server Swiss v.8.05 [16], [17] and the Human pyridoxal phosphate phosphatase (2oycA), that share a similarity of 29%, as template. A Maximum-Likelihood tree was built using MEGA software version 5.1 [18], with a bootstrapping of 1000. Pairwise deletion method was selected for the gap/missing data.

### 2.10. Statistical analysis

Statistical analyses were performed using the GraphPad Prism Software (San Diego, CA, USA). The results are expressed as means ± S.E.M. Significant differences (P<0.05) were determined with a one-tailed student t-test or one-way ANOVA followed by a pair-wise comparison of means (Tukey's test).

## Results

### 3.1. Identification of three A. aegypti FPPases expressed in the CA

**Figure 1:**
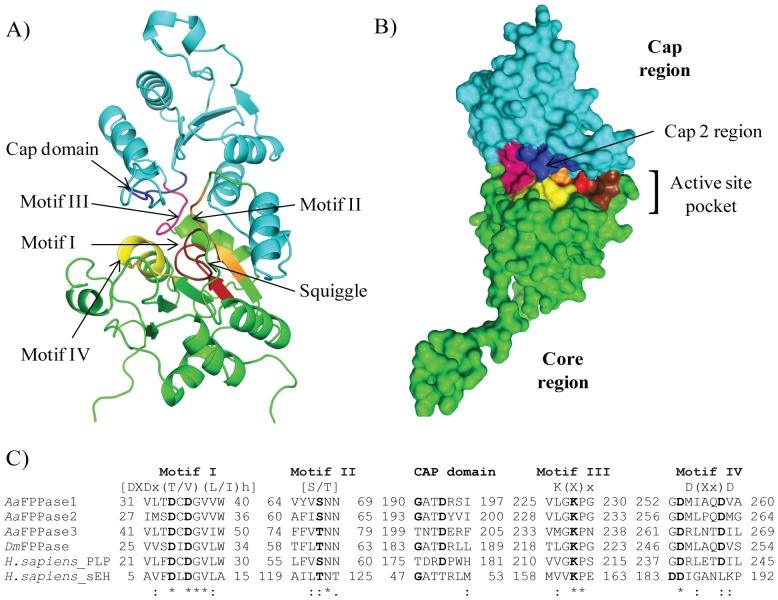
Homology model of the overall fold of *Aa*FPPase-1 and amino acid sequence alignment of HAD motifs and cap domains from mosquito, fruit fly and human. (A) Homology model of the overall fold of *Aa*FPPase-1. Core region is colored in green and cap region in light blue. Motifs are indicated by colors: motif I (red), motif II (orange), motif III (pink), motif IV (yellow), cap domain (dark blue) and squiggle (chocolate). B) Molecular surface diagram illustrating the active site pocket and the cap 2 region of *Aa*FPPase-1. Core region is colored green and cap region in light blue. Motifs are indicated by colors: Motif I (red), motif II (orange), motif III (pink), motif IV (yellow), cap domain (dark blue) and squigle (chocolate). The two structures were constructed by PyMOL using the Human pyridoxal phosphate phosphatase (2oycA) as template. C) Amino acid sequence alignment of HAD motifs and cap domains from mosquito (*Aa*FPPase-1, -2 and -3), fruit fly (*Dm*FPPase), human pyridoxal phosphatase (*H. sapiens*_PLP) and human epoxy hydrolase (*H. sapiens*_sEH). The suggested functions for the motifs are: motif I is required for nucleophilic attack, motif II is responsible for substrate binding, the motif III Lys is required for stabilizing the negative charge of the reaction intermediate together with the Ser/Thr of motif II, motif IV is needed for Mg^2+^ ion binding and the cap domain is involved in substrate recognition. Bold letters indicate the conserved residues in each motif. The numbers represent the amino acid positions in the sequences. “h” denotes a hydrophobic residue and “x” any residue. Accession numbers: *Dm*FPPase (CG15739), *H. sapiens*_PLP (NP_064711.1) and *H. sapiens*_sEH (NP_001243411.1).

Using the sequence of a *D. melanogaster* FPPase (CG15739) that converts FPP into FOL (*Dm*FPPase) [7] we screened the *A. aegypti* genome (Vectorbase) [19]. Eight HAD genes displaying over 48% amino acid sequence similarity were identified (Genbank accession numbers: AAEL012292, AAEL010099, AAEL010098, AAEL007097, AAEL007094, AAEL007098, AAEL007090 and AAEL009503). By examining the temporal and tissue dependent expression of the 8 HAD genes by PCR we identified 3 HADs that were expressed in the CA of adult female mosquito at appropriate times (Genbank: AAEL010099, AAEL007090 and AAEL009503) (Figure S1); we named them *Aa*FPPase-1, *Aa*FPPase-2 and *Aa*FPPase-3 respectively, and were further considered as putative *Aa*FPPases that could be involved in JH biosynthesis. Amino acid sequence alignments of *A. aegypti* and *D. melanogaster* FPPases revealed a number of well conserved residues typical of the HAD phosphatases, including an aspartic acid (Asp_36_) that acts as the catalytic nucleophile, a serine or threonine (Ser_67_) for binding the phosphate group and two aspartic acid residues (Asp_253_, Asp_258_) important for binding the Mg^2+^ cofactor [20], [13] (Fig. 1). The *Aa*FPPase-1 structure obtained by homology modeling exhibited the typical HAD core and cap regions, with the catalytic site as a composite of the four conserved loops of the core region and the loop 5 of the cap region (cap 2 domain) (Fig. 1).

**Figure 2:**
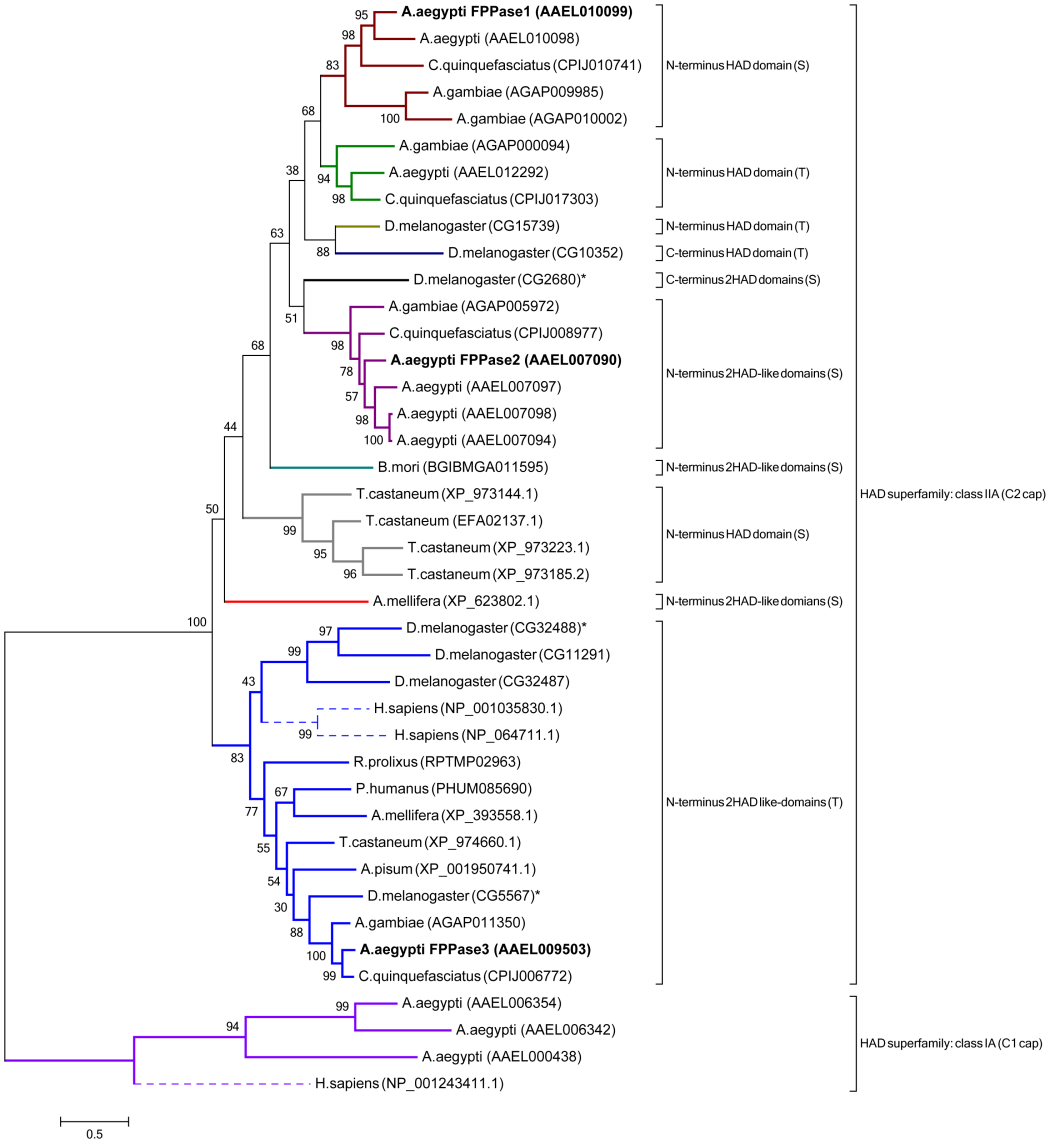
Phylogenetic analysis of HAD superfamily sequences from insects and human. Sequences are labeled with species names and accession numbers in between brackets. The bifunctional human epoxy hydrolase (NP_001243411.1) was used as outgroup. Sequences grouped in two clades. All sequences in Clade 1 are members of the NagD family included in the class IIA of HAD proteins. Sub-clades are separated by the localization of the HAD domain and the presence of a Ser (S) or Thr (T) in motif II. The position of the functional domain is referred as N-terminus or C-terminus. Insects with two potential HAD functional domains are shown with an asterisk. Bold labels represent the *Aa*FPPase- 1, *Aa*FPPase-2 and *Aa*FPPase-3. Human sequences are represented by dotted lines in the tree. All sequences in Clade 2 are epoxy hydrolases, which are members of the class IA of HAD proteins.

A phylogram was generated using FPPase orthologs found in insects and human (Fig. 2). HAD classes IA and IIA clearly separated in two distinct clusters; the main cluster comprises members of the NagD family included in the class IIA with a C2 cap domain (motif V or loop 5) located between the second and third motif. Each of these amino acid sequences contains the conserved four loops (Motif I–IV). Most of the insects phosphatases identified presented one functional HAD domain in the N-terminal of the protein; with many displaying a second incomplete HAD domain in the C-terminus. In addition, three *D. melanogaster* phosphatases had a second functional HAD domain on the C-terminal. We also identified three *D. melanogaster* sequences with a single catalytic HAD domain in the C-terminus of the proteins. Two Human HAD phosphatases (phosphoglycolate phosphatase and pyridoxal phosphatase) were also grouped in the class IIA. Finally, as outgroup we used the bi-functional human epoxy hydrolase that belongs to the Class IA, having a C1 cap located between the motif I and II; this enzyme possess both phosphatase and epoxy hydrolase functional domains. We identified three *A. aegypti* orthologs of the epoxy hydrolase, but they only possess the epoxy hydrolase domain.

### 3.2. All AaFPPases hydrolyzed p-NPP, but only AaFPPase-1 and -2 converted FPP into farnesol

**Figure 3:**
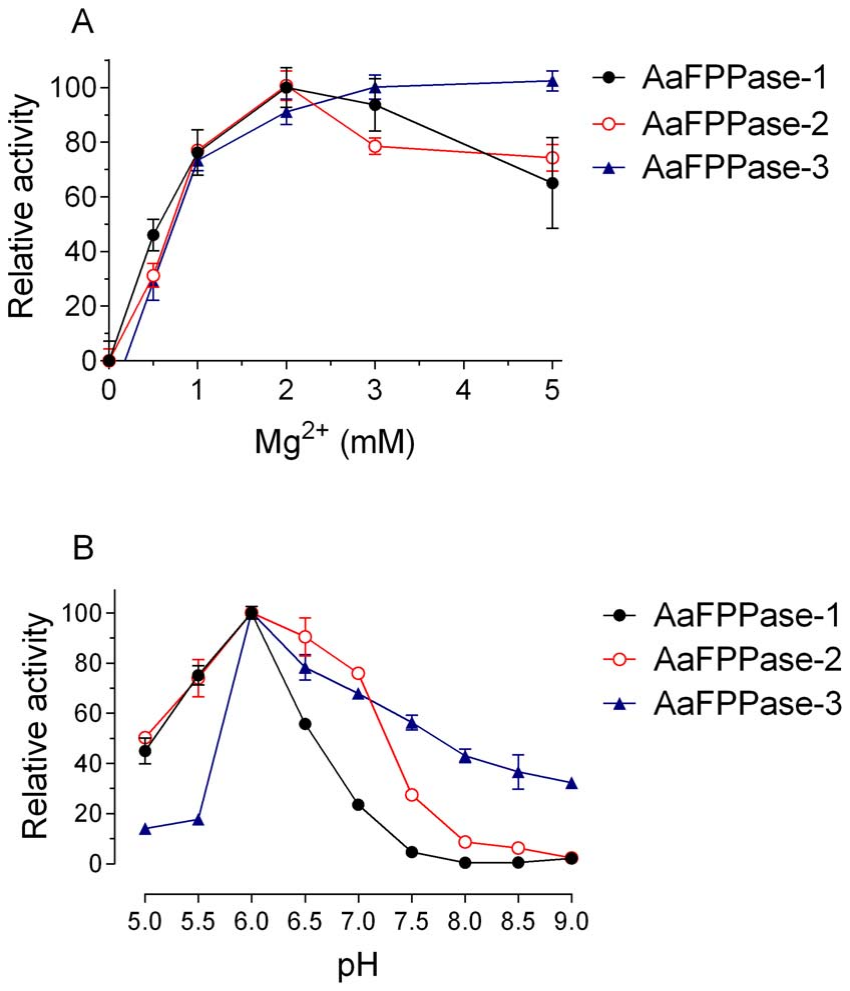
Metal dependence and optimum pH. Phosphatase activity was measured using *p*-NPP. A) Magnesium dose-dependent increases of activities. B) Optimum pH determinations. Three different buffers were used: Sodium acetate at pH 4.5 to 5.5, MES at pH 5.5 to 7 and Tris at pH 7 to 9. Each value represents the means ± S.E.M. of three replicate assays. Relative activity is defined as a percentage of the highest value recorded.

The three putative *Aa*FPPases were overexpressed in *E. coli*. Recombinant His-tagged proteins (∼35 kDa) were purified and phosphatase activities were measured using para-nitrophenyl phosphate (*p*-NPP), a chromogenic substrate for most phosphatases, including alkaline, acid, protein tyrosine and serine/threonine phosphatases. *Aa*FPPase-2 (Km = 315.5±46.9 µM) had higher affinity for *p*-NPP than *Aa*FPPase-1 (Km = 3959.43±126.78 µM). All *Aa*FPPases increased their catalytic activities in a dose-response manner when Mg^2+^ was used as a cofactor (Fig. 3) reaching their maximum activity at pH 6.0 (Fig. 3), which is consistent with previous findings in fruit flies [7].

The specific activities of *Aa*FPPases toward isoprenoid phosphates were measured using the malachite green assay, in which the amount of released inorganic phosphate is determined by quantifying the formation of a complex between malachite green molybdate and free orthophosphate that absorbs at 620–640 nm [21]. Only *Aa*FPPase-1 and *Aa*FPPase-2 efficiently hydrolyzed FPP into FOL (Km = ∼222 µM) (Table 1). *Aa*FPPase-1 (Km = 184.45±14.16 µM) and *Aa*FPPase-2 (Km = 273.98±2.52 µM) also efficiently hydrolyzed GPP. Both enzymes also demonstrated a low affinity for IPP (Table 1). Both enzymes displayed higher “catalytic efficiencies” for GPP than for FPP with Kcat/Km specificity constants for GPP 3–4 fold higher than those for FPP (Table 1). Conversion of FPP into FOL by *Aa*FPPase-1 and *Aa*FPPase-2 was confirmed by RP-HPLC analysis (Figure S2). For the substrates used in this study we found no evidence that pyrophosphate was released from *Aa*FPPases catalyzed reactions. The malachite green phosphate assay does not detect pyrophosphate, but only identifies free phosphate released in solution. In addition, when we treated the products of the *Aa*FPPases catalyzed reaction with pyrophosphatase (an enzyme which cleaves a pyrophosphate into two phosphate ions) we did not detect any significant increase in the amount of free phosphate.

**Table 1:** Substrate specificity for *Aa*FPPase-1 and *Aa*FPPase-2.

Substrate	Km µM ±SE	Vmax µmol min^−1^mg^−1^ ± SE	Kcat s^−1^	Kcat/Km M^−1^s^−1^	Recombinant Enzymes
FPP	222.36±11.0	6.45±0.76	3.33	1.5×10^4^	*Aa*FPPase-1
GPP	184.45±14.16	12.71±0.37	7.92	4.3×10^4^	*Aa*FPPase-1
IPP	>900	ND	ND	ND	*Aa*FPPase-1
FPP	221.02±15.62	5.77±0.15	2.98	1.32×10^4^	*Aa*FPPase-2
GPP	273.98±2.52	28.3±0.95	17.49	6.3×10^4^	*Aa*FPPase-2
IPP	>900	ND	ND	ND	*Aa*FPPase-2

Two isoprenoid-derived compounds, AGGC, AFC and a lipid sulfate were evaluated as potential inhibitors of the *Aa*FPPase catalytic activity. While AGGC was a potent inhibitor of *Aa*FPPase-1 and *Aa*FPPase-2 (Figure S3), AFC and taurolithocholic acid 3-sulfate had little effect.

### 3.3. The CA exhibited variable FPPase activity

**Figure 4:**
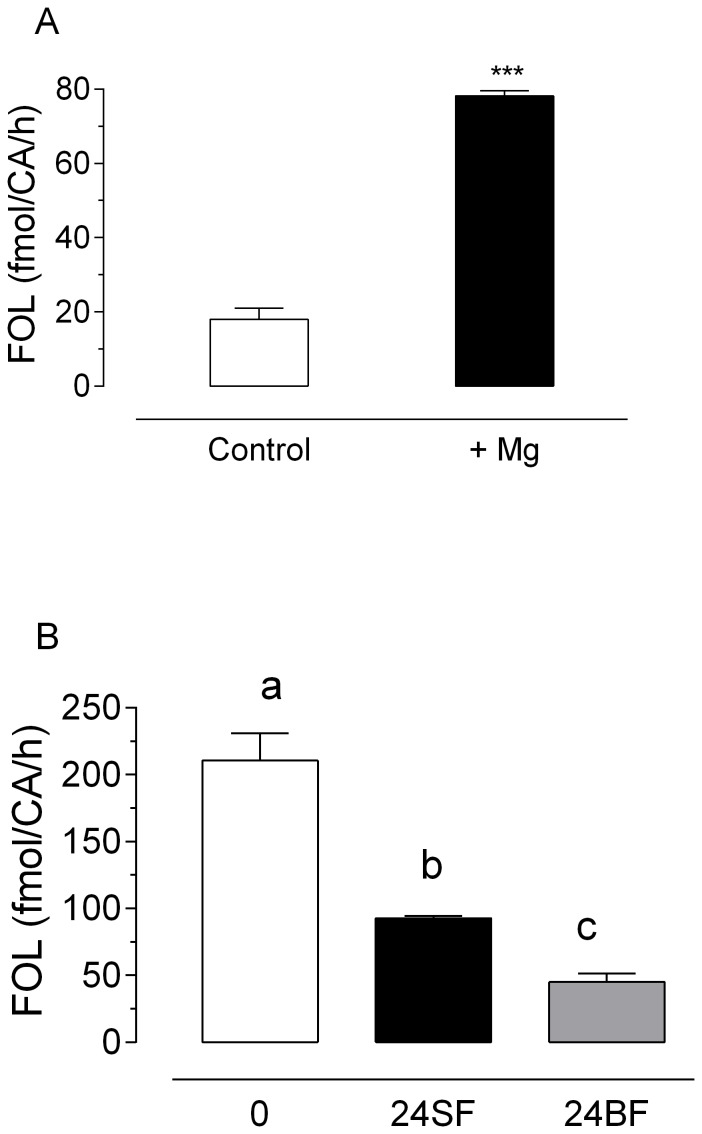
FPPase activity in CA extracts. A) Effect of Mg^2+^ on FPPase activity: Extracts of CA dissected from sugar-fed females 24 h after emergence were incubated with or without 2 mM MgCl_2_. Bars represent the means ± S.E.M. of three replicates of extracts from groups of 5 CA. Asterisks denote significant difference (unpaired t-test, ***P<0.001). B) The CA exhibited variable FPPase activity: Extracts of CA dissected from newly emerged females (0), 24 h after emergence (24SF) and 24 h after blood feeding (24BF) were incubated for 1 h in the presence of an excess of FPP. Bars represent the means ± S.E.M. of three replicates of extracts from groups of 10 CA. Different letters above the columns indicate significant differences among treatments (one way ANOVA p<0.05, with Tukey's test of multiple comparisons).

*Corpora allata* extracts were able to convert FPP into FOL, with the FPPase catalytic activity increasing more than 4 fold when 2 mM MgCl_2_ was added (Fig. 4A). *Aa*FPPase activities were measured in CA extracts from adult female mosquitoes having three distinct JH biosynthetic conditions: basal activity (0 h or newly emerged adult), high activity (24 h sugar-fed) and suppressed activity (24 h after blood feeding). In the presence of an excess of FPP, highly active glands produced 92 fmol of FOL/CA/h, while suppressed glands produced only 45 fmol of FOL/CA/h. The CA with basal activity from newly emerged females, that produced only 12 fmol/h of JH, had quite elevated FPPase activity (210 fmol of FOL/CA/h) (Fig. 4B).

### 3.4. Tissue- and developmental-stage-specific expression of AaFPPases

**Figure 5:**
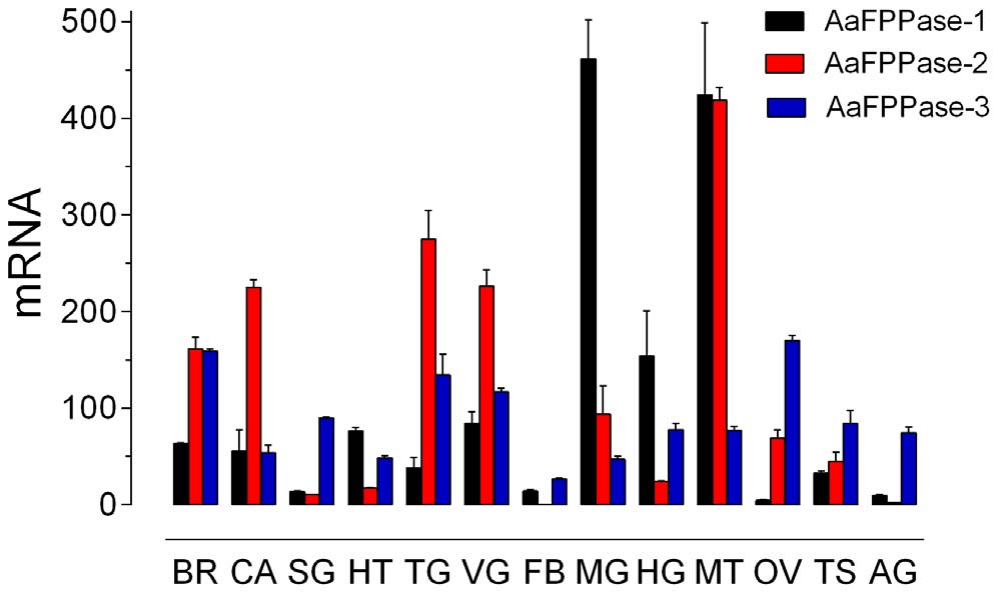
Tissue specific expression of *Aa*FPPases. All tissues were dissected from 3-day-old sugar-fed females, except for testis and accessory glands dissected from 3-day-old sugar-fed males. BR: brain; CA: corpora allata; SG: salivary gland; HT: heart; TG: thoracic ganglia; VG: ventral ganglia; FB: fat body; MG: midgut; HG: hindgut; MT: Malpighian tubules; OV: ovaries; TS: testis and AG: accessory gland. Each value represents the means ± S.E.M of two independent biological replicates of 10–20 tissue samples evaluated in triplicate. *Aa*FPPase mRNAs are expressed as copy number of mRNA/10,000 copies of rpL32 mRNA.

**Figure 6:**
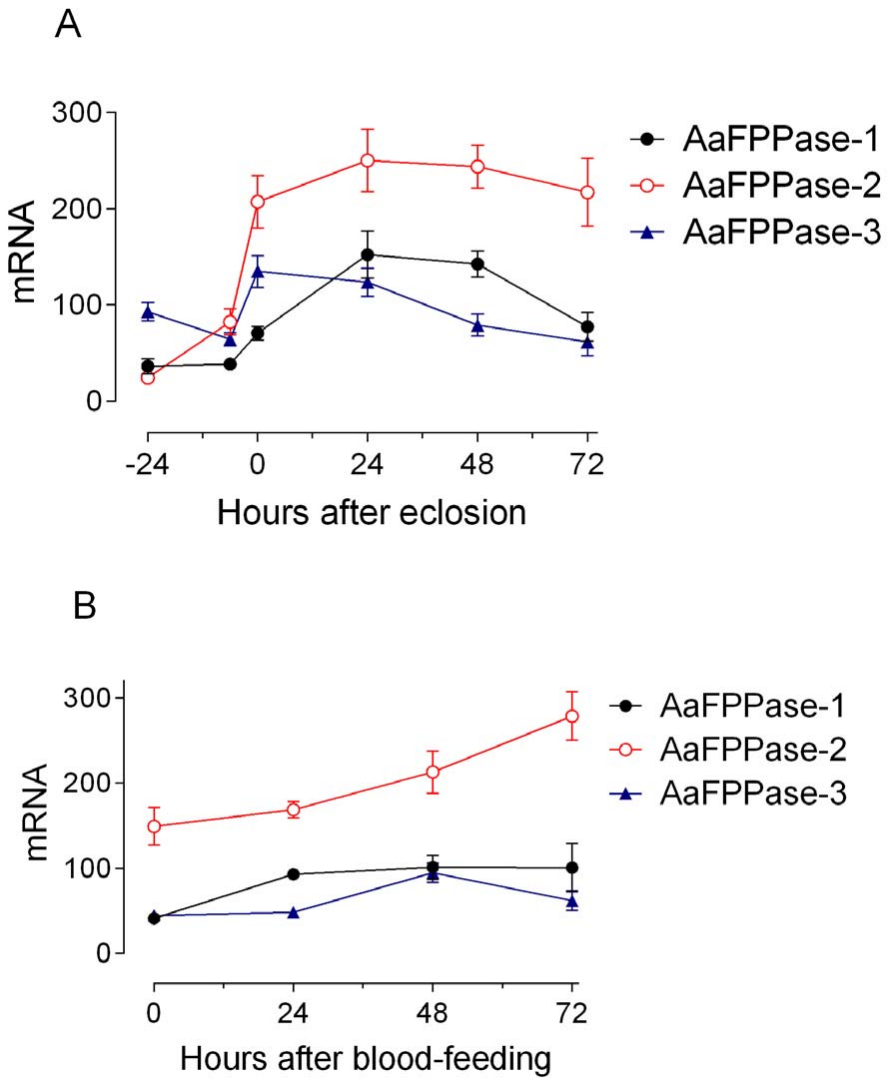
Developmental expression of *Aa*FPPases. A) Expression on pupae and sugar-fed females: mRNA was isolated from CA of pupae 24 h (−24) and 6 h before adult eclosion, newly emerged adult female (0 h), sugar-fed females 24, 48 and 72 h after eclosion. B) Expression after blood feeding. Each data point is the means ± S.E.M. of three independent biological replicates of 20 CA evaluated in triplicate. *Aa*FPPase mRNAs are expressed as copy number of mRNA/10,000 copies of rpL32 mRNA.

Quantitative real time PCR was used to analyze the tissue- and developmental-stage-specific expression of *Aa*FPPases. All three *Aa*FPPase genes were expressed in the CA, but highest transcript levels were detected in other mosquito tissues. The highest level of *Aa*FPPase-1 mRNA was detected in midgut and Malphigian tubules, while that of *Aa*FPPase-2 mRNA in Malphigian tubules and *Aa*FPPase-3 transcripts were most abundant in brain and ovaries (Fig. 5). A developmental time course of mRNA expression in the CA showed that transcripts of *Aa*FPPase-1 and *Aa*FPPase-2 were low in late pupae, increased after emergence and peaked at day one in sugar-fed mosquitoes (Fig. 6A). *Aa*FPPase-3 transcripts levels remained relatively constant for the same period. Transcript levels for the three *Aa*FPPase genes moderately increased after blood-feeding (Fig. 6B).

### 3.5. Reduction of AaFPPase-1 by RNAi caused a significant decrease on JH biosynthesis

**Figure 7:**
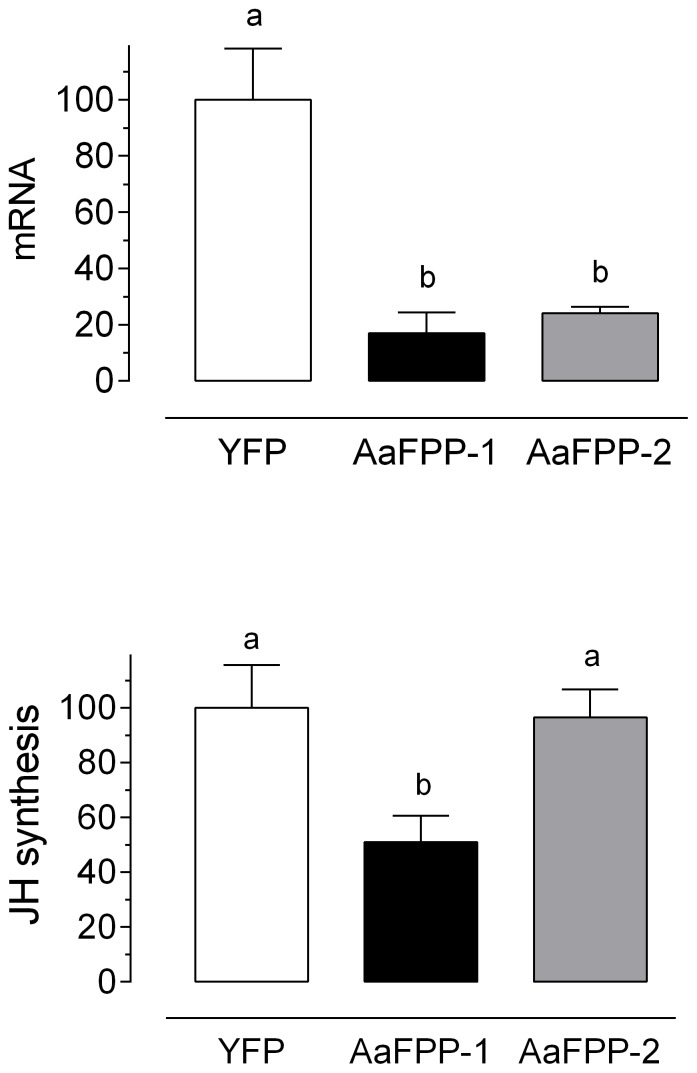
dsRNA mediated knockdown of *Aa*FPPase-1 and -2. Newly emerged female mosquitoes were injected with ds*Aa*FPPase-1, ds*Aa*FPPase-2 or dsYFP; 4 days later transcript and JH levels were evaluated. A) Transcript levels are expressed as % of the YFP controls. Bars represent the means ± S.E.M. of two replicates of RNA extracted from thoraxes. B) JH synthesized *in vitro*: CA were dissected from females injected with ds*Aa*FPPase-1, ds*Aa*FPPase-2 or YFP dsRNA and incubated *in vitro* for 4 h. JH was evaluated by HLPC-FD. Bars represent the means ± S.E.M. of four replicates of 4 CA. Different letters above the columns indicate significant differences among treatments (one way ANOVA p<0.05, with Tukey's test of multiple comparisons).

Since *Aa*FPPase-3 did not appear to catalyze FPP, it was not further considered to have a major role in JH biosynthesis. Therefore the effect of mRNA depletion using RNAi was only studied with *Aa*FPPase-1 and *Aa*FPPase-2. Injection of dsRNA resulted in a significant reduction of *Aa*FPPase-1 and *Aa*FPPase-2 mRNAs (∼80%) (Fig. 7A). Reduction of *Aa*FPPase-1 transcripts resulted in a significant reduction in JH biosynthesis when compared with CA of females treated with dsYFP or ds*Aa*FPPase-2 (Fig. 7B).

## Discussion

### 4.1. Molecular and functional characterization of AaFPPases expressed in the corpora allata of mosquitoes

In this study we had identified and characterized two *corpora allata* mosquito NagD phosphatases that are able to convert FPP into FOL. The homology model of *Aa*FPPase-1 exhibited the typical HAD core and cap regions [22], [13]. The core region is considered to be a modular phosphoryl-transfer unit with the squiggle and flap motifs providing a solvent exclusion mechanism that allows HAD enzymes to alternate between “open” and “closed” conformations. The enzyme in the “open” configuration allows the substrate to enter the active site and interact with the highly conserved catalytic residues in the 4 core motifs and the cap [13], [9]. Upon cap closure, some residues in the cap domain enter the active site and engage in catalysis. Once the substrate is bound, the enzyme assumes a “closed” configuration and the Mg^2+^ ion in the active site interacts with the negatively charged phosphate, preparing it for nucleophilic attack by the first conserved aspartate on motif I [13] (Figure S4).

*Aa*FPPase-1 and the previously described *Dm*FPPase (GC15739) [7] are both expressed in the CA, process FPP into FOL and are part of a cluster of NagD family members that contain one functional active site (HAD domain) in the N-terminus of the core unit. Additional close related NagD sequences in other insects exhibited variability on the number and location of the HAD domains; although the effect of these changes on activity and substrate specificity remains to be studied. The study of FPPases from additional insect species could help to improve our understanding of the basis of isoprenoid phosphate binding specificity in NagD insect proteins.

### 4.2. Expression of AaFPPases genes

Previous studies in *Bombyx mori* [23], [24] and *A. aegypti* [6] suggested that the transcripts for most of the JH biosynthetic enzymes were highly enriched or exclusively expressed in the CA. The last two metabolic reactions, the methylation of FA and the epoxidation of MF, are most likely exclusive for JH biosynthesis and therefore the enzymes involved (juvenile hormone acid methyl transferase and epoxidase) should be highly expressed in the CA [6]. In contrast, other enzymes in the late pathway, such as the *Aa*FPPases described in these studies, farnesol dehydrogenases [3] and farnesal dehydrogenases [4] are broadly expressed in many tissues. This is not surprising since farnesol and farnesal homeostasis are vital for cells in all insect tissues. Farnesol acts as a signaling molecule in cell proliferation and apoptosis [25], [26], [27]. Posttranslational modifications by attachment of a farnesyl group to C-terminal cysteine of target proteins by farnesyl-transferases are essential for signal transduction and vesicular transport [28]. Farnesal dehydrogenases play key roles in the generation of fatty alcohols and fatty acids as well as in the elimination of toxic biogenic and xenobiotic aldehydes, such as those produced by oxidative damage of glycerolipids or during protein deprenylation [29], [30], [31]. The presence of more than one isozyme capable of catalyzing the hydrolysis of long chain pyrophosphates in mosquitoes suggests that selection mechanism caused duplication and diversification of members of the NagD family and facilitated the evolution of more efficient substrate specificities, as well as a better tissue and developmental regulation; essential for the critical role that these phosphatases play in every cell.

### 4.3. AaFPPase-1 and JH biosynthesis

JH levels must be modulated to enable the normal progress of development and reproductive maturation in mosquitoes [32]. Changes in JH titers in female adult *A. aegypti* mosquitoes are very dynamic. The CA needs to adjust its synthetic activity to generate these dynamic changes [33]. The rate of JH biosynthesis is controlled by the rate of flux of isoprenoids in the pathway, which is the outcome of a complex interplay of changes in precursor pools, enzyme levels and external regulators [6]. Changes in the nutritional status in female mosquitoes [34], as well as the manipulation of individual precursor pool concentrations (e.g. FOL, FAL and FA) affect the rate of JH biosynthesis [6]. Dynamic changes of JH biosynthesis are controlled in part by a coordinated expression of the enzymes associated to the pathway [6]. Using an HPLC-fluorescence approach, we were able to measure the changes in the production of FOL by *Aa*FPPase from CA extracts dissected from newly emerged mosquitoes, sugar-fed and blood-fed female mosquitoes. As was shown with the recombinant proteins, the FPPase activity of the CA extracts were Mg^2+^ dependent, and exhibited remarkable differences among basal, highly active and depressed glands. In sugar-fed females, we found a good concordance between *Aa*FPPase-1 and -2 mRNA expressions in the CA and JH biosynthesis [33]. Although the highest transcript levels of *Aa*FPPases were found in highly active glands, the maximum enzyme activity was found in basal active glands, suggesting that the molecular basis for JH regulation is quite unique at different times during the reproductive cycle of an adult female mosquito.

We have previously described a 1000-fold difference in the levels of mRNA expression in the CA among the JH biosynthetic enzymes [6]. Four enzymes presented overall low levels of expression, acetoacetyl-CoA thiolase, phosphomevalonate kinase, farnesol dehydrogenase and farnesal dehydrogenase [6], [4]; transcripts numbers for *Aa*FPPase-1 are also low and comparable to the levels of those 4 genes. Under some conditions any of these enzymes could become rate limiting or “bottleneck”. We have reported that the low enzymatic activity of farnesal dehydrogenase could be a restrictive factor for JH biosynthesis in the CA of blood-fed mosquitoes [4]; a similar condition might apply to *Aa*FPPase-1, the decrease in enzymatic activity detected after blood-feeding might reduce the farnesol pool to levels that could limit the flux of precursors and JH biosynthesis.

*Aa*FPPase-1 and -2 efficiently hydrolyzed FPP into FOL. Therefore, we selected these 2 genes for RNAi studies Although the RNAi mediated silencing was efficient for both enzymes, we found JH biosynthesis was significantly reduced only in *Aa*FPPase-1 silenced mosquitoes CA, suggesting that *Aa*FPPase-1 is predominantly involved in JH biosynthesis.

### Conclusions

A search for orthologs of a farnesyl phosphatase described in *D. melanogaster* led to the identification of two NagD *Aa*FPPases that are expressed in the CA of *A. aegypti* and efficiently hydrolyzed FPP into FOL. A combination of RNAi experiments and biochemical studies using CA extracts and recombinant proteins support the hypothesis that these HAD enzymes convert FPP into FOL in the CA and might be involved in JH biosynthesis in mosquitoes.

## Supporting Information

Figure S1 **PCR analysis of the expression of eight putative phosphatase genes in the CA of adult female** ***Aedes aegypti*** **.**
(PDF)

Figure S2 **Chromatogram of a reverse-phase high performance liquid (HPLC) analysis showing the production of farnesol from FPP by** ***Aa*** **FPPase-1.**
(PDF)

Figure S3 **Effect of the inhibitor AGGC on** ***Aa*** **FPPase activity.**
(PDF)

Figure S4 **Schematic representation of the catalytic mechanism for** ***Aa*** **FPPases.**
(PDF)

Table S1 **Primers used for RT-PCR, Q-RT-PCR, and production of dsRNA**.
(PDF)
