# Supplementary material for: Farnesyl Phosphatase, a Corpora allata Enzyme Involved in Juvenile Hormone Biosynthesis in Aedes aegypti
Source: PLoS One. 2013 Aug 5;8(8):e71967. doi: 10.1371/journal.pone.0071967 (PMC3734299; doi:10.1371/journal.pone.0071967)

Figure S1: PCR analysis of the expression of eight putative phosphatase genes in the CA of adult female *Aedes aegypti*. cDNA was made from: (1) CA dissected at the time of adult emergence, (2) one day-old sugar-fed females. From left to right: AAEL012292, AAEL010099 (*Aa*FPPase-1), AAEL010098, AAEL007097, AAEL007094, AAEL007098, AAEL007090 (*Aa*FPPase-2) and AAEL009503 (*Aa*FPPase-3).

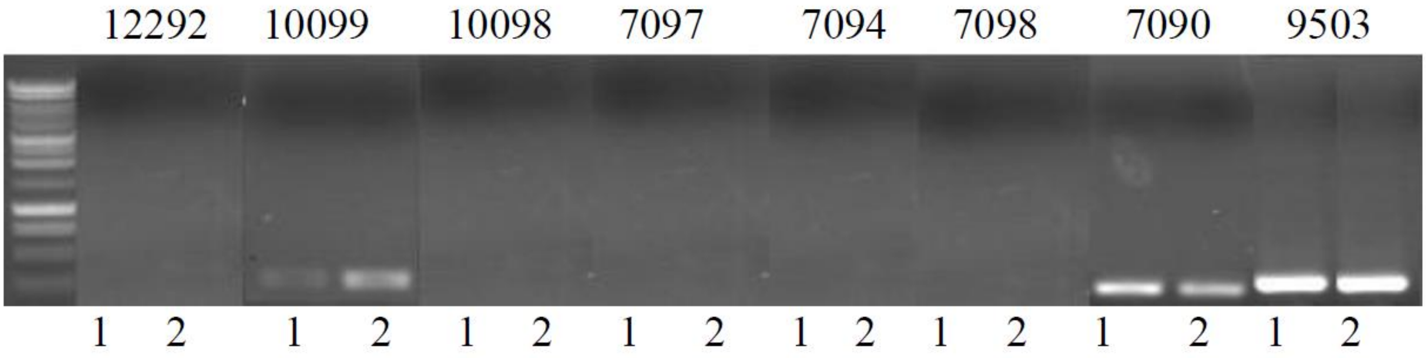

Supplement: Figure S1 — PCR analysis of the expression of eight putative phosphatase genes in the CA of adult female Aedes aegypti . (PDF) [file pone.0071967.s001.pdf]
