# Supplementary material for: Farnesyl Phosphatase, a Corpora allata Enzyme Involved in Juvenile Hormone Biosynthesis in Aedes aegypti
Source: PLoS One. 2013 Aug 5;8(8):e71967. doi: 10.1371/journal.pone.0071967 (PMC3734299; doi:10.1371/journal.pone.0071967)

Figure S2: Chromatogram of a reverse-phase high performance liquid (HPLC) analysis showing the production of farnesol from FPP by *Aa*FPPase-1. A) 300µM FPP was incubated with *Aa*FPPase-1 in reaction buffer for 1hr at RT. Arrow indicates farnesol (retention time 37.5 min). B) Negative control in which 1mM FPP was incubated in reaction buffer without adding enzyme for 1hr at RT.

A

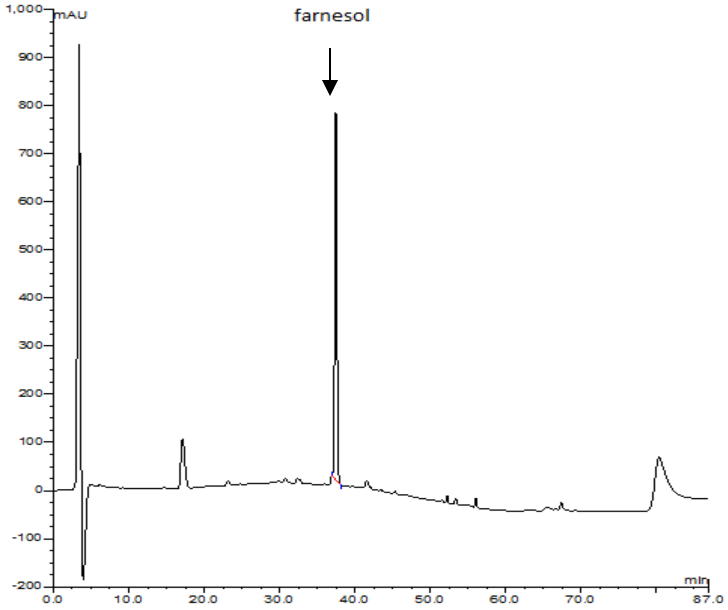

B

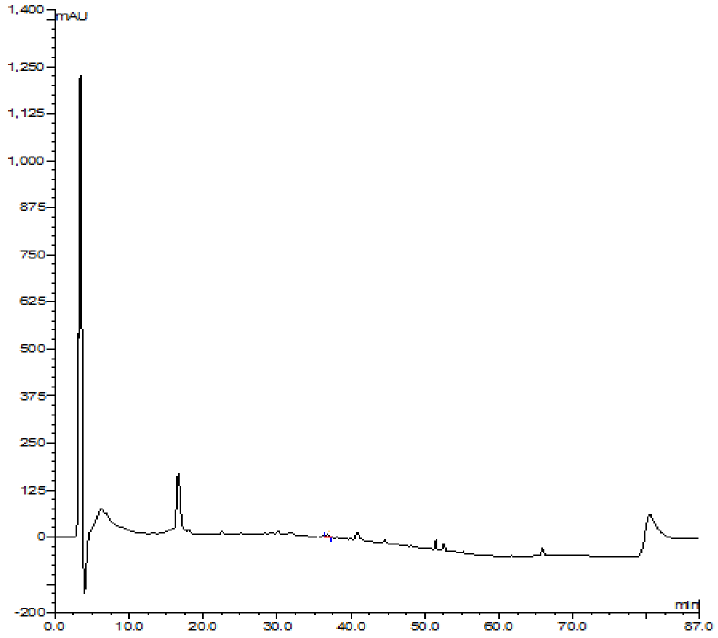

Supplement: Figure S2 — Chromatogram of a reverse-phase high performance liquid (HPLC) analysis showing the production of farnesol from FPP by Aa FPPase-1. (PDF) [file pone.0071967.s002.pdf]
