# Supplementary material for: Farnesyl Phosphatase, a Corpora allata Enzyme Involved in Juvenile Hormone Biosynthesis in Aedes aegypti
Source: PLoS One. 2013 Aug 5;8(8):e71967. doi: 10.1371/journal.pone.0071967 (PMC3734299; doi:10.1371/journal.pone.0071967)

Figure S3. Effect of the inhibitor AGGC on *Aa*FPPase activity: Recombinant *Aa*FPPase-1 and -2 were pre incubated with different concentrations (0 to 40 μM) of N-acetyl-S-geranylgeranyl-L-cysteine (AGGC) for 10 min and their activities were measured using the *p*-NPP assay.

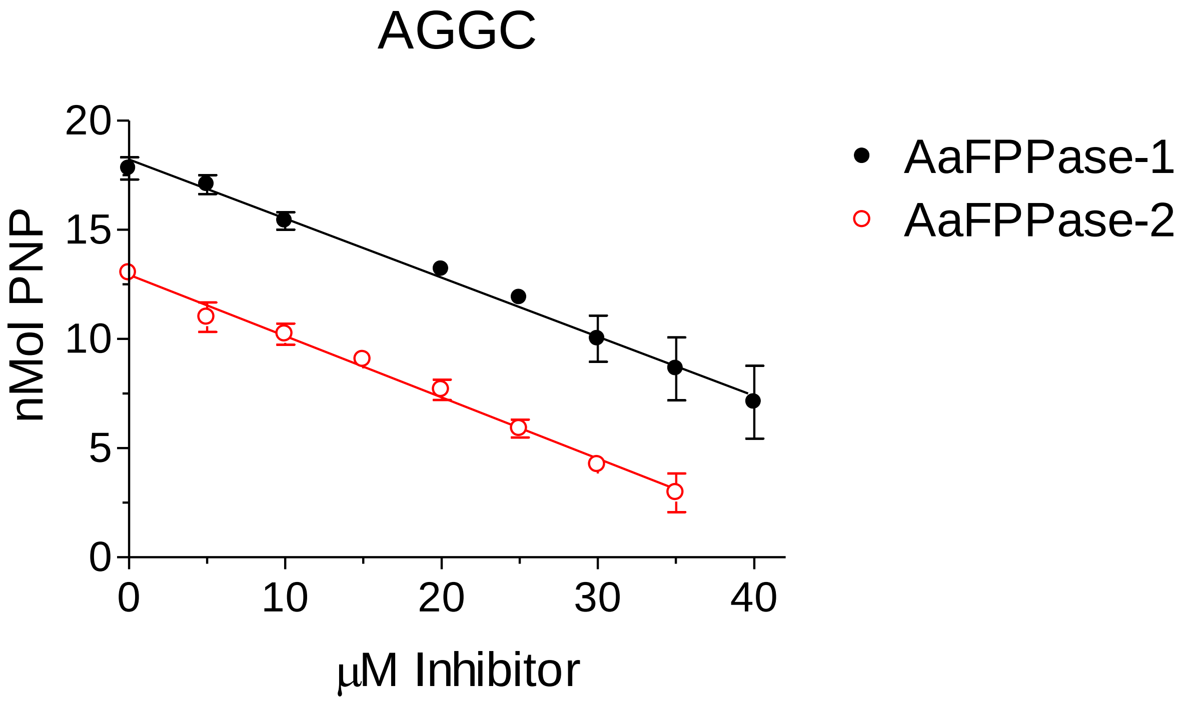

Supplement: Figure S3 — Effect of the inhibitor AGGC on Aa FPPase activity. (PDF) [file pone.0071967.s003.pdf]
