# Supplementary material for: Farnesyl Phosphatase, a Corpora allata Enzyme Involved in Juvenile Hormone Biosynthesis in Aedes aegypti
Source: PLoS One. 2013 Aug 5;8(8):e71967. doi: 10.1371/journal.pone.0071967 (PMC3734299; doi:10.1371/journal.pone.0071967)
Supplement: Figure S4 — Schematic representation of the catalytic mechanism for Aa FPPases. (PDF) [file pone.0071967.s004.pdf]

Figure S4. Schematic representation of the catalytic mechanism for *Aa*FPPases:

Catalysis proceeds through an aspartylphosphate intermediate. **1)** Once the FPP is bound, the Mg^{2+} ion in the active site interacts with the negatively charged phosphate, preparing it for nucleophilic attack by the first conserved aspartate on motif I. **2)** As a result, an acyl phosphate intermediate is formed with the carboxyl group of this aspartate and a water molecule is deprotonated by the second aspartate of motif I; hydrolyzing the acyl phosphate intermediate and returning the enzyme to the native state **3)** The enzyme forms a new complex with FMP. **4)** Catalysis of FMP occurs again through an aspartylphosphate intermediate. **5)** Farnesol is released and the enzyme returns to the initial state.

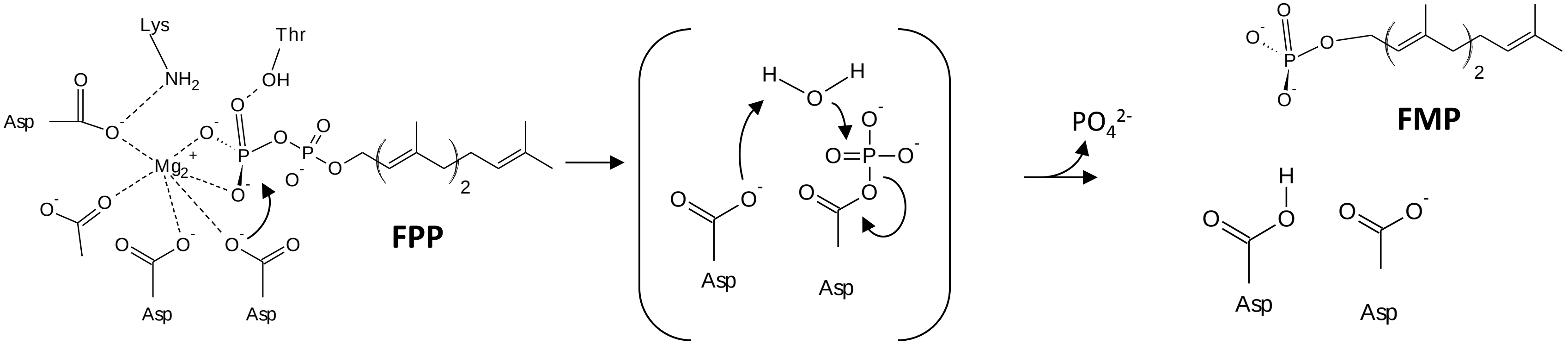

❶ Enzyme –substrate complex. Nucleophilic attack by Asp on the phosphoryl group.

❷ Phosphoaspartyl enzyme-intermediate. Nucleophilic attack by water and release of the free phosphate.

❸ Free enzyme. Enzyme returns to initial state.

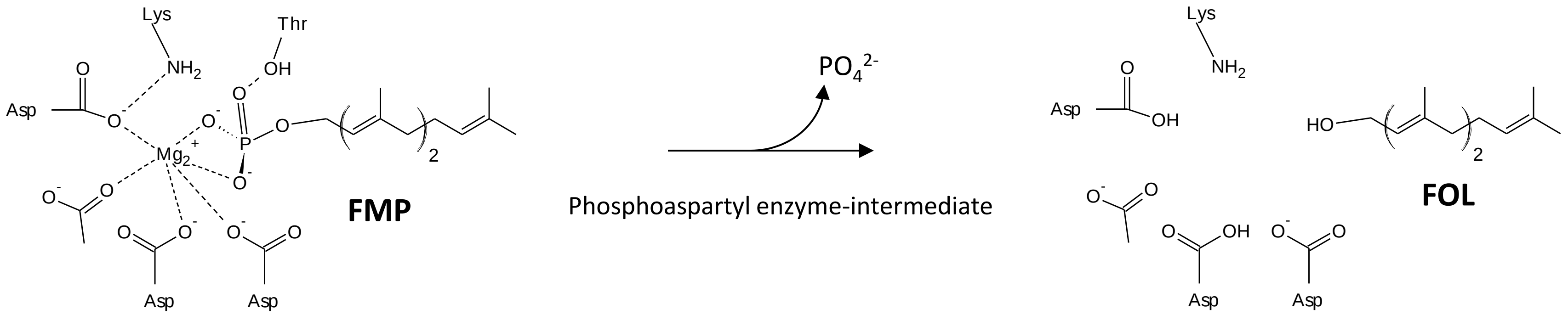

❹ Enzyme-substrate complex.

❺ Free enzyme. Enzyme returns to initial state.
