# Supplementary material for: Farnesyl Phosphatase, a Corpora allata Enzyme Involved in Juvenile Hormone Biosynthesis in Aedes aegypti
Source: PLoS One. 2013 Aug 5;8(8):e71967. doi: 10.1371/journal.pone.0071967 (PMC3734299; doi:10.1371/journal.pone.0071967)
Supplement: Table S1 — Primers used for RT-PCR, Q-RT-PCR, and production of dsRNA. (PDF) [file pone.0071967.s005.pdf]

Table S1

Primers used for RT-PCR

Gene accession number	Forward Primer	Reverse Primer
AAEL012292	5’CGTTGATTCGTTCGATTGTG 3’	5’CGAATGTACGAACGCTGTTG 3’
AAEL010099	5’GAGGACGTCGTTCATCCAGT 3’	5’AATCTACCACCACCGCTTTG 3’
AAEL010098	5’TTCGAGGGTTTGATCTACGC 3’	5’GAATGCACTCGGGATCACTT 3’
AAEL007097	5' TCTCCGAAACGAGCAGTACA 3'	5' GGATTCCGTTCCAGATAGCA 3'
AAEL007094	5’ GTCGTGAGGCTTTGGTTCTC 3’	5’AATTCTTCGGGCTTGTTGTG 3’
AAEL007098	5’ GTCGTGAGGCTTTGGTTCTC 3’	5’ AATTCTTCGGGCTTGTTGTG 3’
AAEL007090	5’ TTGGGACGGAGGTGTTTAAG 3'	5' GGCTTTCATGAGATGGGACA 3'
AAEL009503	5’ CATCGAGAATGGGAAGCAGT3’	5’ GGAACCGACCACGTACACTT 3’

Primers used for Q-RT-PCR

Primer	Sequence
rpL32 F	5’ CCATCAGTCCGATCGCTATGA 3’
rpL32 R	5’ GTTGTCAATACCTTTCGGCTTACG 3’
rpL32 Probe	5’ CAAGCTTGCCCCCAACTG 3’
AaFPPase-1 F	5’ AGGGATGCAGGGTTTGAAGTTATTC 3’
AaFPPase-1 R	5’ GATAAGACGGAGCGATTCTGGTT 3’
AaFPPase-1 Probe	5’ ATGGGCCGAACGATGCA 3’
AaFPPase-2 F	5’ GGAGGTGTTTAAGAACTATCTACGATCA 3’
AaFPPase-2 R	5’ GGCGGCGCCTCCAT 3’
AaFPPase-2 Probe	5’ CCGTTGGCCCATCTAG 3’
AaFPPase-3 F	5’ GTGGCCAAATCGGTGAAACTG 3’
AaFPPase-3 R	5’ GCAGCTAGATAGGCGGTAGAGATAA 3’
AaFPPase-3 Probe	5’ TTGTCAACGCCAACATT 3’

Primers used for production of dsRNA

AaFPPasei-1 F	5’ AGTTATTCATGGGCCGAACGATGC 3’
AaFPPasei-1 R	5’ ATATCGACCCGAGCCCAGAACTTCAA 3’
AaFPPasei-2 F	5’ CGATCAGAAGGATTTACTGTTCTAG 3’
AaFPPasei-2 R	5’ CAGCAAACAGTCAGGATTGC 3’
YFPi F	5’ AACCGCATCGAGCTGA 3’
YFPi R	5’ ATGGTCAGGCGGGACT 3’
AaFPPasei-1 FT7	5’ TAATACGACTCACTATAGGGAGTTATTCATGGGCCGAACGATGC 3’
AaFPPasei-1 RT7	5’TAATACGACTCACTATAGGGATATCGACCCGAGCCCAGAACTTCAA3’
AaFPPasei-2 FT7	5’ TAATACGACTCACTATAGGGCGATCAGAAGGATTTACTGTTCTAG 3’
AaFPPasei-2 RT7	5’ TAATACGACTCACTATAGGGCAGCAAACAGTCAGGATTGC 3’
YFPi FT7	5’ TAATACGACTCACTATAGGGAACCGCATCGAGCTGA 3’
YFPi RT7	5’ TAATACGACTCACTATAGGGATGGTCAGGCGGGACT 3’
